# Perceiving temporal structure within and between the senses: A multisensory/crossmodal perspective

**DOI:** 10.3758/s13414-025-03045-2

**Published:** 2025-04-28

**Authors:** Nicola Di Stefano, Charles Spence

**Affiliations:** 1 Institute of Cognitive Sciences and Technologies, Via Gian Domenico Romagnosi 18A, 00196 Rome, Italy; 2 Crossmodal Research Laboratory, University of Oxford, Oxford, UK

**Keywords:** Crossmodal, Multisensory, Rhythm, Metre, Temporal pattern

## Abstract

The literature demonstrates that people perceive temporal structure in sequences of auditory, tactile, or visual stimuli. However, to date, much less attention has been devoted to studying the perception of temporal structure that results from the presentation of stimuli to the chemical senses and/or crossmodally. In this review, we examine the literature on the perception of temporal features in the unisensory, multisensory and crossmodal domains in an attempt to answer, among others, the following foundational questions: Is the ability to perceive the temporal structure of stimuli demonstrated beyond the spatial senses (i.e., in the chemical senses)? Is the intriguing idea of an amodal, or supramodal, temporal processor in the human brain empirically grounded? Is the perception of temporal structure in crossmodal patterns (even) possible? Does the ability to perceive temporal patterns convey any biological advantage to humans? Overall, the reviewed literature suggests that humans perceive rhythmic structures, such as beat and metre, across audition, vision and touch, exhibiting similar behavioural traits. In contrast, only a limited number of studies have demonstrated this ability in crossmodal contexts (e.g., audiotactile interactions). Similar evidence within the chemical senses remains scarce and unconvincing, posing challenges to the concept of an amodal temporal processor and raising questions about its potential biological advantages. These limitations highlight the need for further investigation. To address these gaps, we propose several directions for future research, which may provide valuable insights into the nature and mechanisms of temporal processing across sensory modalities.

## Introduction

Perceiving the temporal structure of sensory stimuli is a highly complex ability that is crucial to many aspects of perception and adaptive behaviour across several species, including humans (for reviews, see Fitch, 2012; Kotz et al., 2018; Ravignani et al., 2014). Temporal structuring refers to the ability to extract perceptual patterns/features that apply to a sequence of rapidly presented separate sensory stimuli. While such an ability is clearly related to the perception of time, and especially to duration discrimination, it is more refined, allowing, for example, for the extraction of hierarchical structures (i.e., the beat or metre) from trains of pulses presented to the ears, eyes, or skin.

Besides being crucial for the perception of repetitive patterns in a wide variety of perceptual contexts, from music to literature and poetry, temporal organization is key to many cognitive tasks that are predicated on a higher-level recognition of the structural ordering of stimuli. Moreover, beyond perceptual and cognitive domains, the perception of temporal features, such as rhythm and isochrony, has been shown to have important cascading effects on the development of social behaviours and creating social bonding across individuals (e.g., Rabinowitch & Knafo-Noam, 2015; Trainor & Cirelli, 2015).

The perception of temporal structure in sequences of stimuli is a general ability that can be experienced in several different sensory modalities (for early sources, see Fraisse, 1948, 1981; Isaacs, 1920; Ruckmick, 1913, 1917, 1927). People naturally perceive the rhythm of auditory stimuli, as well as of tactile pulses or flashing lights. The ability to perceive temporal structure in sequences of stimuli across different sensory modalities has also been considered important for human development. Lewkowicz (2000) has long argued for the central importance of temporal stimulus features in facilitating infant multisensory development. In particular, according to the ‘intersensory redundancy hypothesis’ (IRH), the temporal qualities of stimuli, such as isochrony and rhythm, provide a framework for establishing relationships between sensory features that are coded differently in each sense (e.g., see Bahrick & Lickliter, 2004; Lickliter & Bahrick, 2012). This is because temporal information is putatively available across the senses, creating a common point of reference. For example, in infants, the rhythm of a caregiver’s rocking motion (kinesthetic sense) often synchronizes with the soothing tone of their voice (auditory sense), thus allowing the two senses to synchronize and the infant to integrate the information seamlessly. According to Lewkowicz (2003), initially in development, infants attend to the overall temporal structure of rhythmic audiovisual events and only later (e.g., by 10 months) do they become aware of synchrony (see also Parise et al., 2012, 2013, on the fundamental importance of temporally correlated signals to multisensory integration).

In this tutorial review, we provide a thorough examination of humans’ ability to perceive temporal structuring in sequences of stimuli in a range of sensory modalities and in the context of crossmodal/multisensory perception. The focus is primarily on rapidly presented stimuli that repeat in a short timescale only, i.e., presentation rates that allow the observer to clearly distinguish between pulses within a train of stimulation (ranging from ∼200 to 2,000 ms; London, 2004). We do not focus on timing per se – which is a much broader topic encompassing a wide range of mechanisms and phenomena across various timescales – nor on the perception of the temporal order of stimuli presented sequentially to different senses – which pertains primarily to the sequencing of stimuli (e.g., determining “before” or “after”) and does not engage with the structural aspects of perception that arise from rhythmic or isochronous patterns. This focused approach allows us to provide a more in-depth discussion on how temporal structure is perceived and processed.^1^

We start by analysing the perception of rhythm, metre and beat, in musical sounds before turning our attention to address the question of whether similar temporal grouping (or organizational) principles, or Gestalts (e.g., Tenney & Polansky, 1980; cf. Notter et al., 2019), are also present in the visual and tactile modalities. Thereafter, we take a closer look at the literature documenting crossmodal influences of the temporal structure present in one modality (normally audition) on the perceived temporal structure present in a different modality (typically vision or touch). Much of the literature that has been published to date is consistent with the view that audition dominates the other senses in the perception of temporal organization, be it in terms of flicker/flutter, repetition rate, rhythm, metre, or beat. This leads to a summary of the literature on the perception of multisensory temporal structure (i.e., where the equivalent temporal information is presented simultaneously to different senses).

Thereafter, we try to answer the question of whether it is even possible to experience such perceptual phenomena when the temporal organization of stimuli emerges as a result of stimulation taking place in more than one sensory modality. That is, where the temporal pattern, or structure, is not present in the input delivered to any one of the senses when considered individually. The search for such crossmodal phenomena, referred to as ‘inter-sensory Gestalten’ (by Gilbert, 1938), as ‘transmodal Gestalts’ (by Kubovy & van Valkenburg, 2001), and as ‘crossmodal Gestalts’ (by Spence, 2015; Spence & Di Stefano, 2025) has thus far uncovered surprisingly few robust examples. The latter absence is nevertheless consistent with the view that intramodal perceptual grouping typically occurs much earlier in time (Cook & Van Valkenburg, 2009), and often totally dominates over any crossmodal perceptual organizational, especially when sequences of stimuli are presented in each modality (for reviews, see Spence, 2015; Spence et al., 2007). Furthermore, such observations have (controversially) been taken by others to argue against awareness, or consciousness, being, in any meaningful sense, multisensory (Spence & Bayne, 2015). They can also be taken to argue against the suggestion that the perception of rhythm is amodal, as has been claimed by a number of researchers over the years (for a review, see Spence & Di Stefano, 2024a). We assess whether the literature published to date supports the existence of an amodal, or supramodal, ability that might be responsible for temporal processing across the senses or, at least, across the modalities of audition, touch and hearing. Finally, we try to answer the question of whether and how perceiving temporal patterns might convey a biological advantage, or have any biological role, for humans.

### Structure of the review

The paper is structured as follows: In Sect. "On the perception of temporal structure in sequentially presented unisensory patterns", we provide an extensive review of the multidisciplinary literature on the perception of temporal structure in sequentially presented patterns in the unisensory domain. The section starts with a preliminary overview of discrimination thresholds for perceiving distinct pulses in sequences of stimuli in audition, vision and touch. Thereafter, in Sect. "Crossmodal influences of the perception of temporal patterns presented to different senses", we consider the literature on the crossmodal influences of the perception of temporal patterns presented to different senses. Sect. "On the perception of temporal structure in crossmodally presented patterns" deals with the fundamental question of the perception of crossmodal structure in temporal patterns, that is, the perception of an emergent temporal organization that is not present or perceptible in any of the constituent sequences of unisensory stimuli. In Sect. "Discussion", we discuss a number of outstanding theoretical issues, related to topics such as amodality, synchrony, multisensory integration, and the potential biological advantages associated with the ability to perceive temporal organization across the senses. We conclude our review by outlining some novel directions for future empirical research in the perception of temporal patterns across the senses (Sect. "Conclusions").

## On the perception of temporal structure in sequentially presented unisensory patterns

In this section, we take a closer look at the literature that has investigated humans’ perception of temporal structure in sequences of musical sounds (Sect. "Perceiving the temporal structure in sequences of sounds"), where the key notions of beat and metre were first elaborated. Thereafter, we move to comparing the perception of temporal structures in sequential unimodal auditory, visual and tactile stimuli (Sect. "Comparing the perception of temporal structures in sequential unimodal auditory, visual and tactile stimuli"), taking a closer look at those studies that have investigated whether and how humans perceive rhythmic elements, such as beat and metre, beyond the musical and more generally the auditory domains. Before reviewing these studies, we first provide an overview of the temporal discrimination thresholds related to the perception of distinct pulses in a succession of stimuli across different sensory modalities in Sect. "Temporal discrimination thresholds for the perception of sequences of stimuli in audition, vision and touch".

### Temporal discrimination thresholds for the perception of sequences of stimuli in audition, vision and touch

The range of tempi over which rapidly presented sequences of stimuli in different sensory modalities are perceived as separated is limited (Fraisse, 1963, 1982). As far as audition is concerned, the evidence suggests that the gap detection threshold is in the order of 2–3 ms for clicks and noises, while it ranges from 6–9 to 17 ms for sinusoidal tones depending on the frequencies, i.e., the lower the frequency, the higher the threshold (for a review, see Merchel & Altinsoy, 2020). Studies indicated 10 Hz as a conservative estimate for the fastest frequency for successive sounds to be clearly distinguished (Friberg & Sundström, 2002; London, 2004). Since thresholds are affected by the level of stimulation, different values might depend on slightly different features of the stimuli delivered during the discrimination task, such as dynamic versus static (see Mikkelsen et al., 2020, on the reproducibility of detection/discrimination thresholds with vibrotactile stimuli).^2^

In early psychophysical research, Talbot and colleagues (1968) reported that, for touch, the delivery of sine-wave mechanical stimuli feels like a light flutter of the skin in the frequency range between 5–40 Hz, while from 40–60 to 250 Hz the sensation turns into a vibratory hum, which is harder to localize (for a review, see Romo & Salinas, 2003). As far as the interstimulus interval (ISI) is concerned, Piéron (1952, pp. 296–297) suggested that the discrimination threshold to perceive two neighbouring touches as distinct stimuli is 10 ms. Similar values (8–12 ms) have also been reported by Merchel and Altinsoy (2020; for a systematic review, see also Silva et al., 2025).

In the visual domain, Piéron (1952, pp. 296–297) indicated 100 ms as the minimum gap between successive visual stimuli to be clearly perceived as distinct, although these values vary according to luminance, spatial separation, intensity and other features of the stimuli (see Boynton, 1972, for a review). As far as regards the minimum duration of the stimuli, Cheatam and White (1952) found that flashes of 11-ms duration (separated by 88 ms) were most accurately perceived by all participants as compared to faster presentations/shorter durations.

Gault and Goodfellow (1938) extended the Seashore auditory discrimination test (Seashore, 1938) to three sensory modalities. Specifically, participants were presented with pairs of unimodal rhythmic patterns and asked to discriminate whether they were the same or different. The percentage of correct responses was 84.9% for hearing, 74.8% for vision, and 70.4% for touch, evidencing a higher accuracy of hearing with respect to vision and touch. However, the discrimination thresholds for the visual, auditory and tactile modalities were found to be the same and in the order of 20 ms (Hirsh & Sherrick, 1961). The lower accuracy of tactile discrimination was later confirmed by Van Erp and Werkhoven (2004), who proved that the length of temporal intervals is systematically overestimated in touch compared to visually presented intervals. Gescheider (1966, 1967) found that the minimum ISI for two auditory stimuli to be perceived as discrete is 2 ms, while for tactile stimulations is 10–12 ms (see also Occelli et al., 2011).

Factors affecting the ability to perceive rapidly presented stimuli as a sequence of distinct stimuli include the duration of each stimulus and the interstimulus interval.^3^ Those values are very much affected by the physiological properties of the sensory system, i.e., photoreceptors, auditory fibres, or the type of skin receptors stimulated.^4^ However, as a general rule, for stimuli to be perceived as separated, shorter stimulus durations must be compensated for by longer ISIs. That being said, the perception of temporal patterns is likely going to be limited by the respective limitations in the temporal limits of information processing affecting the various senses individually (see Gallace et al., 2012; Zimmerman, 1989; see Table 1).

**Table 1:** Minimum duration and interstimulus interval values for audition, vision and touch

Sense	Physical nature of stimulus	Minimum duration of stimulation to consciously perceive that a discrete stimulus has been presented	Minimum interstimulus interval (ISI)
*Audition*	Mechanical waves	20–50 ms (sinusoidal tones)	2 ms (clicks and noises) 6–17 ms (sinusoidal tones)
*Vision*	Electromagnetic waves	10–30 ms	50–100 ms
*Touch*	Mechanical waves (for vibrotactile sensations)	25–50 ms	10 ms

The minimum duration refers to the shortest amount of time the sensory system needs to be stimulated to consciously perceive that a stimulus has been presented. The interstimulus interval is the minimum temporal gap required between two successive stimuli for them to be perceived as separate. Note that these values can vary significantly as a function of the measurement methods (e.g., method of limits, method of constant stimuli, method of adjustments) and specific parameters of the stimuli, such as body location for touch, brightness for vision, and loudness or frequency for audition

Having made these introductory remarks, it is now time to summarize the literature on the perception of temporal structure in unisensory auditory stimulus sequences.

### Perceiving the temporal structure in sequences of sounds

Audition is the prototypical sensory domain in which people can extract temporal features from sequentially presented stimuli. Hirsh (1967, p. 22) stated that any theory of auditory perception requires “the concepts of sequence and temporal pattern to play the same role that Gestalt or form or shape has played in visual perception”. An extensive body of evidence has shown that human sensitivity to purely temporal information is better in the auditory than in the visual modality (e.g., Goodfellow, 1934; Grondin, 1993; Grondin & Rousseau, 1991; Grondin et al., 1998, 2001; Rousseau et al., 1983). When it comes to motor reproduction of, or synchronization to, temporal patterns, humans perform better when the stimuli are perceived auditorily (Gault & Goodfellow, 1938; Glenberg & Jona, 1991; Glenberg et al., 1989; Repp & Penel, 2004).

Although temporal organization can be perceived across various categories of auditory stimuli, such as speech and noise (e.g., Navarra et al., 2014; Poeppel & Assaneo, 2020; Smit et al., 2024), we will first examine the case of sound/music perception.^5^ This is because it likely represents the prototypical context in which repetitive and regular stimuli are perceived and categorized. Moreover, the definition of rhythmic structures and elements (e.g., beat or metre) is originally formulated within the context of music. However, many principles that apply to the perception of temporal structure of sound/music also extend, with some distinctions, to the perception of speech and noise (Patel, 2003).

As highlighted by Bizley and Cohen (2013), temporal regularity is key to the perception of auditory objects, conceived as “the computational result of the auditory system’s ability to detect, extract, segregate and group the spectrotemporal regularities in the acoustic environment into stable perceptual units” (Bizley & Cohen, 2013, p. 693).^6^ When dealing with musical sounds, such temporal regularity is often referred to as ‘rhythm’. Through rhythm, temporality works as a principle of the organization of musical elements, for example, notes, phrases, passages and sections. Moreover, rhythm is used as a means by which to create hierarchies amongst sounds, therefore structuring, organizing and forming the architecture of music composition. Hereinafter, we provide an operational definition of rhythm (Sect. "Rhythm perception") and of its constituent notions of beat (Sect. "Beat perception") and metre (Sect. "Metre perception"), reviewing some key findings in the empirical literature on auditory perception.

#### Rhythm perception

In music theory and music perception, while the notions of beat and metre are apparently clear-cut (see below), a “precise, generally accepted definition of rhythm does not exist” (Fraisse, 1982, p. 149). According to McAuley (2010), the term rhythm is ambiguously used in music to refer either to the sound pattern or to the perception of that pattern. With respect to the sound pattern, rhythm is the sequence of durations of a series of events; for example, the rhythm of a melody is the serial pattern of durations marked by sounds (notes) and silences (rests). With respect to perception, rhythm refers to the perceived temporal organization of the physical sound pattern (i.e., the series of notes and rests).

**Fig. 1:**
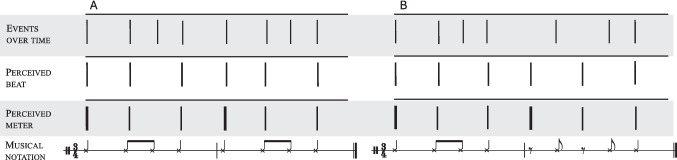
Schematic representation of rhythmic structures. The perceived beat is a regular pulse that is extracted from a sequence of stimuli, even when they are not isochronous. Notably, the perception of a beat does not always correspond to an actual auditory input, as demonstrated by the syncopated rhythm in the right panel. Metre helps organize temporal events hierarchically through the alternation of strong (thicker lines) and weak (thinner lines) accents

In rhythmic sequences, several types of structure can be discerned (see Fig. 1). First, humans perceive patterns of different temporal intervals (or interstimulus intervals). Second, humans might be able to extract a regular, periodic beat in response to a rhythm. The beat is not always directly associated with the actual presence of an auditory input, thus highlighting that it is a perceptual rather than a stimulus feature (see Fig. 1B). Rhythms can contain hierarchical metrical structure, with the salience of events depending on their temporal ordering (e.g. ‘metre’, such as alternating strong and weak beats in a march). For example, the same six isochronous beats can be organized into a march rhythm, in which every other beat is accented (**1** 2 **1** 2 **1** 2), or into a waltz, in which the first of every three beats is accented (**1** 2 3 **1** 2 3). Finally, rhythmical patterns can be written in musical notation.

The ability to perceive rhythm develops very early in humans. For instance, infants as young as 2 months of age have been shown to be able to discriminate simple rhythmic patterns that have contrasting successive patterns of duration (e.g., Chang & Trehub, 1977; Lewkowicz, 2003), and they do this even in the presence of concurrent changes to the pitch level and tempo of rhythms (Trehub & Thorpe, 1989). For example, Demany et al. (1977) demonstrated that infants (1.5–3 months of age) differentiate between a continuous sequence of identical 40-ms auditory stimuli separated by 194-ms intervals (i.e., 194–194-194–194 ms), and another sequence of the same sounds having the identical duration with different spacing (194–97–194–291 ms). The ability to discriminate between more complex rhythmic structures is evident in 5-month-old (Chang & Trehub, 1977) and also in 7-month-old infants (Allen et al., 1977). Comparing the performance of adults and infants, Trehub and Hannon (2009) examined the detection of subtle rhythmic and melodic changes to two sequences of tones, a conventional rhythm that musically untrained adults rated as rhythmically good and an unconventional rhythm that was rated as poor. Their findings revealed that both adults and infants performed more precisely in the context of the conventional rhythm.

Regular or temporally predictable rhythms are typically preferred and more easily processed than irregular or less predictable rhythms. For example, when listening to isochronous rhythms with different tempi, adults readily differentiate them on the basis of their tempo, but performance declines significantly when adults listen to non-isochronous rhythms (Drake & Botte, 1993). These findings can be explained in terms of temporal attention or temporal expectancy, that is, claiming that attention is directed to the point in time that is predicted by perceived rhythms (e.g., Vuust et al., 2018; though see Ahmad et al., 2024, on the effect of short-term explicit learning on people’s perception of non-isochronous Indian metres).^7^

Interestingly, besides playing a crucial role in the perception of harmonic features, such as consonance and dissonance (see Di Stefano et al., 2022), simple ratios have also been shown to affect the perception of rhythm.^8^ In particular, non-isochronous rhythms are perceived as more regular if their component durations are related by simple ratios, such as 1:1 and 2:1 (Essens & Povel, 1985; Keller & Repp, 2005). When reproducing rhythms (e.g., with clapping or finger-tapping), humans are more accurate when the component durations are related by simple-integer ratios than by complex- or non-integer ratios (Essens, 1986; Essens & Povel, 1985; Povel & Essens, 1985; Sakai et al., 1999).

Neurophysiological evidence confirms that the neural representation of a rhythm depends on its interval ratio. For instance, the participants in a study by Sakai and colleagues (1999) performed a short-term memory task for a seven-tone rhythm sequence, which was formed with 1:2:4, 1:2:3, or 1:2.5:3.5 ratios. Behavioural data confirmed that the reproduction of more regular rhythms (1:2:4 and 1:2:3) was more accurate than the reproduction of the irregular rhythm (1:2.5:3.5). Moreover, neurophysiological findings (functional magnetic resonance imaging (functional magnetic resonance imaging, fMRI) indicated that the brain activation patterns for 1:2:4 and 1:2:3 rhythms were quite similar but were completely different from that for 1:2.5:3.5 rhythm. The left premotor and parietal areas and right cerebellar anterior lobe were active for 1:2:4 and 1:2:3 rhythms, whereas the right prefrontal, premotor and parietal areas together with the bilateral cerebellar posterior lobe were active for 1:2.5:3.5 rhythm. These results suggested that there are two neural representations for rhythm depending on the interval ratio, corresponding to metrical and non-metrical representations, respectively.^9^

#### Beat perception

Most forms of music, either written or not (e.g., improvised), are organized by (quasi-) isochronous pulses. Beat perception is a cognitive ability that allows for the detection of these regular pulses, i.e., beat, in music (Honing, 2013; Large & Palmer, 2002).^10^ In addition to creating a regular pacing that influences listeners’ processing of auditory stimuli, for example, through temporal and structural expectation (e.g., Large & Jones, 1999; Large & Palmer, 2002; Meyer, 1960), such an ability is considered at the basis of one of the most universal behaviours that music typically induces in listeners, i.e., sensorimotor synchronization to music (e.g., through finger or foot taps, or body sway; for reviews, see Repp, 2005; Repp & Su, 2013).

As beat perception is seemingly absent in spoken language (e.g., Patel, 2008), it might be considered a domain-specific skill. Interestingly, humans do not need special training to perceive and entrain to musical beat; rather it appears to be a robust and ubiquitous behaviour.^11^ Some authors have suggested that the ways in which babies are rocked and bounced in time to music by their parents is the most important factor in developing this sense for metrical structure (cf. Trehub & Hannon, 2006). By contrast, more recent studies have emphasized a biological basis, showing that beat induction is already functional in young infants (Zentner & Eerola, 2010) as well as in 2- to 3-day-old babies (Winkler et al., 2009).

In a recent EEG-MMR (Electroencephalography-Mismatch Response) study, Haden and colleagues (2024) compared mismatch responses of infants’ brains to infrequent deviants falling on either accented or unaccented (i.e., odd and even) positions. The results revealed a clear difference between responses to metrical positions in the isochronous sequence, but not in the equivalent jittered sequence. These results support Winkler et al.’s (2009) conclusion that beat detection is already functional at birth in healthy infants, seemingly implying that beat detection (at least in newborns) is unlikely to be mediated by statistical learning occurring after birth. In contrast, these findings could be explained by considering the auditory experiences of the fetus, which may establish a biological predisposition to perceive temporal regularity, as experienced in the womb through rhythms like the mother’s heartbeat or respiration (for a review, see Ullal-Gupta et al., 2013; see also Ivanov et al., 2009, on maternal–fetal heartbeat synchronization).

Neurophysiological studies have revealed an increase in basal ganglia activity associated with the perception of rhythms that induced a beat compared with similar rhythms that did not induce any beat (Grahn & Brett, 2007; Grahn & Rowe, 2009). The role of basal ganglia in beat perception has been indirectly confirmed by studies showing that Parkinson’s disease patients (for whom basal ganglia function is impaired) exhibit impaired discrimination abilities when presented with the same beat-inducing rhythms (Grahn & Brett, 2009). In addition to the basal ganglia, supplementary motor area (SMA) activation is also associated with both perception and production of beat (for a review, see Leow & Grahn, 2014).

#### Metre perception

If beat is the perception of regular pulses, metre is the perception of a hierarchical differentiation of pulses according to the alternation of strong and weak accents (Apel, 1972; Cooper & Meyer, 1960). Although the notion of metre has primarily been applied to, and investigated in, the context of music, the perception of metre-like accents can be elicited by isochronous and unaccented pulse trains too (Bolton, 1894; Woodrow, 1909).^12^

Metrical processing begins early in life. According to the results of one event-related potential (ERP) study, newborns differentially process events occurring at strong versus weak metrical positions (Winkler et al., 2009). Further developmental studies have confirmed that older infants are sensitive to changes in metre. For example, when presented with rhythms with different metres, 7-month-old infants were able to discriminate rhythms that violate those metres they were familiar with (Hannon & Johnson, 2005).

Cultural exposure to music is likely to affect the ability to perceive metre. Research shows that Western listeners have difficulty in perceiving, remembering, reproducing and tapping synchronously to rhythmic patterns containing metres that they are not typically exposed to (Essens, 1986; Essens & Povel, 1985; Fraisse, 1982; Hannon & Trehub, 2005; Repp et al., 2005; Snyder et al., 2006). By contrast, individuals from Turkey and India, who are accustomed to non-isochronous metres, do not exhibit enhanced perception and production of 2:1 over 3:2 ratios (Hannon & Trehub, 2005; Hannon et al., 2012).

The presence of a clear metre strongly affects listeners’ perception of rhythm and other features of sounds. Bharucha and Pryor (1986) demonstrated that rhythms that can present a metric hierarchy are more easily discriminated than those rhythms that do not fit within a metric framework. Additionally, Jones et al. (1982) demonstrated that it is harder for listeners to discriminate pitches occurring at metrically weak locations than those occurring at metrically strong ones. The evidence reviewed in this section demonstrates that the influence of metre on auditory perception extends beyond rhythm, affecting how listeners process various sound attributes, including pitch, within a structured temporal framework.

### Comparing the perception of temporal structures in sequential unimodal auditory, visual and tactile stimuli

In an early study by Handel and Buffardi (1968), participants had to identify temporal patterns composed of stimuli presented to different sensory modalities, namely, audition, touch and vision. For each modality, two dichotomous elements were generated, namely, a 1,200-Hz tone and a 3,000-Hz tone for audition, two vibrotactile stimuli one held in each hand (6 V, 30 Hz and 12 V, 60 Hz, respectively), a red and a green panel light for vision. A pattern was composed of a sequence of elements, such as low tone – low tone – low tone – high tone, for audition, and red light – red light – red light – green light for vision (note that the original patterns consisted of eight elements).

In one of the experimental conditions, the entire sequence of eight elements was fully presented first in one modality, then in another. Three pairs of modalities were used: auditory-tactile, auditory-visual, and tactile-visual. The participants were exposed to the stimuli until they could identify the pattern. The results showed that temporal patterns presented in the auditory and tactile modalities were easier to identify than those presented in the visual modality, thus suggesting better temporal pattern perception and recognition in audition and touch compared to vision (see also Handel & Buffardi, 1969).

Marks (1987) conducted a study in which participants had to make similarity judgements concerning pairs of temporal patterns that were either presented in the same or different modalities (hearing, vision and touch). The temporal patterns varied in pulse duration and length of the interval between successive pulses. The findings demonstrated that the perception of pattern similarity remains strikingly uniform regardless of the modality stimulated. In the years since the publication of Marks’ study, a number of studies using a wide variety of crossmodal tasks (comparison/matching) of a temporal sequence presented in one modality on a subsequently presented temporal sequence presented in either the same or different modality have been published (e.g., Allen et al., 1977; Collier & Logan, 2000; Grondin & McAuley, 2009; Guttman et al., 2005; Jokiniemi et al., 2008; Kang et al., 2018).

For instance, Allen and colleagues (1977) had two groups of infants (mean age of 6 months) repeatedly presented with a standard visual or auditory temporal sequence during a habituation period. Then, in the test phase, each group was divided into four subgroups in which the presentation modality and/or the temporal sequence remained the same or were different. Physiological parameters, namely heart rate and galvanic skin conductance, were recorded from each participant during habituation and test phases. The results revealed that infants in the intersensory presentation conditions (auditory-visual and visual-auditory groups) showed greater recovery of both heart rate and skin potential responses to the different temporal sequence than they did to the same temporal sequence. The authors interpreted this finding as evidence of infants’ ability to perceive the equivalence of information across sensory modalities.

Collier and Logan (2000) tested a similar hypothesis by having their participants match two rhythmic sequences either within or across the senses. In a same-different task, pairs of brief rhythms were presented in which each rhythm was either presented visually or auditorily, resulting in two unimodal conditions (visual-visual; auditory-auditory) and two multisensory (auditory-visual; visual-auditory) conditions. Three different rates of presentation were used. The results supported the temporal advantage of the auditory modality in short-term memory, which was quite robust at the fastest presentation rates. This advantage tended to decay as the presentation rate was slowed down, consistent with the view that, with time, the temporal patterns were being recoded into a more generic form.

Using the same experimental paradigm as Collier and Logan (2000), Jokiniemi and colleagues (2008) presented two rhythmic patterns to their participants (N = 12) sequentially. In half of the trials, the rhythms were identical, while in the remainder they were different. The participants had to decide whether the two patterns were the same or different. The patterns were presented in the auditory, tactile and visual modalities. The results revealed that the auditory condition had the highest rate (79.2%) of correct responses, followed by tactile (75.0%) and visual (< 65.0%) (see also Handel & Buffardi, 1968).^13^

In a study by Kang et al. (2018), participants were presented with sequences of acoustic pulses for audition, motion pulses to the fingertips for touch, or light pulses for vision. Pulses were randomly and irregularly spaced, with all inter-pulse intervals in the sub-second range and all constrained to be longer than the minimal temporal duration that can be detected in any individual sensory modality. The results revealed that, if a random temporal pattern re-occurred at random times during an experimental block, it was rapidly learned, whatever the sensory modality. Moreover, and importantly, patterns first learned in the auditory modality displayed transfer of learning to either touch or vision. This suggests that sensory systems may be exquisitely tuned to incidentally learn re-occurring temporal patterns, with possible cross-talk between the senses.

In a seminal experiment on the perception of tactile rhythm, Brochard and colleagues (2008) provided critical evidence for the ability of humans to extract temporal periodicity (i.e., the beat) from purely tactile sequences. The participants in this study were presented with identical rhythmic sequences of events either via the auditory or tactile modality and were asked to synchronize finger tapping to the inferred beat of each sequence. The results confirmed that participants were able to abstract the metric structure from tactile rhythmic sequences as efficiently as from equivalent auditory patterns. Interestingly, almost every participant reported having felt the pulse of most of the tactile sequences.

Studies on sensorimotor synchronization with the beat demonstrated better performance in humans with sequences of auditory and tactile stimuli than with visual flashing sequences (Patel et al., 2005; Repp & Penel, 2004; Varlet et al., 2012), thus mirroring the findings on the primacy of audition in tasks involving temporal perception. In a recent study, Whitton and Jiang (2023) used separate visual, auditory or tactile stimuli to create a metronomic beat with a tempo of 100 bpm, and an ISI of 600 ms. The participants were instructed to tap the space bar on the keyboard with their dominant index finger in synchrony with the steady metre (tempo) defined by flashes presented on the monitor (visual trials), tones presented via headphones (auditory trials), or vibrations delivered on their left index finger (tactile trials). The results showed that sensory-motor synchronization to external rhythmic stimuli was significantly more precise for auditory and tactile than for visual sequences. Interestingly, the findings revealed a correlation between participants’ performance and their musical background.^14^

Johnson et al. (2020) investigated whether trimodal (i.e., audio-visual-tactile) stimuli may yield additional performance benefits in sensorimotor synchronization tasks. The results of the study replicated performance improvements with bimodal compared with unimodal stimuli. However, noteworthily, trimodal stimuli yield less, or in some cases no advantage compared with bimodal stimuli. These results suggest that in the case of trimodal stimulation, the increase of the amount of sensory information does not lead to a linear increase of synchronization performance.^15^

Auditory dominance has also emerged from studies targeting memory. For instance, in Glenberg and colleagues’ (1989) study, rhythms were produced by sequences of short and long auditory stimuli or short and long visual stimuli and the participants were instructed to reproduce the temporal sequence. The results from four experiments demonstrated that the reproduction of auditory rhythms is superior to that of visual rhythms. The modality of reproduction also appears to show preferential mappings. For example, beyond the modality in which temporal sequences happen to be presented, there is also the modality of responding that has been reported to exert an influence over the reproduction of rhythms (Kolers & Brewster, 1985). According to Kosonen and Raisamo (2006), people experience greater difficulty in accurately reproducing rhythms that have been presented visually than tactually, which in turn is worse than the reproduction of auditory rhythms.

#### Neurophysiological evidence

One question that arises is whether the neural substrates responsible for the perception of temporal features, such as beat, are specific to the auditory modality. Although beat perception certainly appears to occur more readily with auditory stimuli, the role of the basal ganglia networks in beat perception might not be specific to the auditory modality. When a visual rhythm is presented after the same rhythm is presented auditorily, a sense of beat can be induced for the visual rhythm, the basal ganglia response increases during the visual rhythm presentation, and the amount of that increase predicts whether a beat is perceived in the visual rhythm (McAuley & Henry, 2010).

Araneda and colleagues (2017) used fMRI to test the extent to which the neural network involved in beat processing is supramodal (or amodal, though see Spence & Di Stefano, 2024a), that is, is the same in the different sensory modalities. Brain activity changes in 27 healthy volunteers were monitored while they were attending to the same rhythmic sequences (with and without a beat) in audition, vision and the vibrotactile modality. A common neural network for beat detection was found in the three modalities that involved parts of the auditory dorsal pathway. Within this network, only the putamen and the SMA showed specificity to the beat, while the brain activity in the putamen covaried with the speed of beat detection. These results highlighted the involvement of the auditory dorsal stream in beat detection, confirming the important role played by the putamen in beat detection, and indicating that the neural network supporting beat detection is mostly supramodal. This constitutes a novel example of convergence of the same functional attributes into one centralized representation in the brain.

Another fMRI study compared brain responses with visual rhythms presented either after or before similar auditory rhythms to examine the difference between visual rhythms that were perceived to have a beat and those that were not. Activity in the basal ganglia during the presentation of visual rhythm significantly predicted whether that visual rhythm induced a beat (Grahn et al., 2011). Overall, these findings suggest that an internal representation of the beat formed during auditory presentation can influence the perception of beats in subsequent visual rhythms, with the basal ganglia playing a key role in mediating this process.

Hove et al. (2013) investigated whether the differences in timing-related brain activation during sensorimotor synchronization tasks with auditory beats and visual flashes reflect differences in tapping synchronization stability or between modality (i.e., audio-motor vs. visuo-motor) integration. Participants synchronized their finger taps with four types of visual and auditory pacing sequences: flashes and a moving bar, as well as beeps and a frequency-modulated ‘siren’. Behavioural tapping results showed that visuo-motor synchronization improved with moving targets, whereas audio-motor synchronization degraded with frequency-modulated sirens. fMRI results showed that activation in the putamen, a key timing area, paralleled the behavioural results: putamen activation was highest for beeps, intermediate for the continuous siren and moving bar, and was lowest for the flashes. Putamen activation differed between modalities for beeps and flashes, but not for the novel moving bar and siren (see also Hoddinott & Grahn, 2024, for results demonstrating activity in the putamen and SMA during beat perception in audition).

By dissociating synchronization performance from sensory modality, Hove and colleagues’ (2013) study demonstrates that activation in the basal ganglia is associated with sensorimotor synchronization stability rather than modality. In conclusion, this study presents evidence that sensorimotor synchronization is largely contingent upon the stimuli’s suitability to the processing style of each modality.

In a similar vein, the study by Bernard and colleagues (2022) investigated whether the experience of rhythm is shared between audio and haptic perception. Using a surface-haptic device designed to synthesize arbitrary audio-haptic textures, they conducted a series of psychophysical studies demonstrating that the perception threshold curves of audio and haptic rhythmic gradients are the same. The findings demonstrated the interaction of both audio and haptic modalities below 60 Hz. Importantly, multisensory integration was documented when the audio and haptic rhythmic gradients were congruent. Such findings suggest that audio and haptic signals are also likely to be processed by common neural mechanisms for the perception of rhythm.

### Interim summary

The research reviewed in this section supports the existence of similar grouping principles operating in audition, vision and touch (see Table 2). Marks (1987) demonstrated that the perception of pattern similarity remains consistent across the spatial senses, while other studies (e.g., Allen et al., 1977; Kang et al., 2018) revealed that temporal patterns learned in one sensory modality can be recognized when presented in other modalities. Moreover, the literature shows that humans can extract the beat from nonauditory sequences of stimuli (e.g., tactile pulses; Brochard et al., 2008). When a visual rhythm is presented after the same rhythm has been presented auditorily, a sense of beat can be induced for the visual rhythm (McAuley & Henry, 2010), possibly by relying on an internal representation of the beat that was formed during the auditory presentation. However, studies consistently show superior sensorimotor synchronization to the beat for auditory and tactile stimuli compared to visual stimuli (Repp & Penel, 2002, 2004). Intriguingly, neuroimaging studies (e.g., Araneda et al., 2017) have shown that beat perception involves a supramodal network, including the basal ganglia and SMA, which is active when an observer is exposed to rhythmic stimuli across auditory, visual and tactile modalities.

**Table 2:** Schematic comparison of the concepts of rhythm, beat and metre across the senses and regarding their contribution to the perception of temporal structure

Concept	Definition	Sensory Modalities	Contribution to Temporal Structure Perception
*Rhythm*	A pattern of durations and intervals between events	Auditory, visual, tactile	Provides the sequential structure of events, organizing them into patterns that can be repeated or varied
*Beat*	A regular, isochronous, and repeating pulse that underlies rhythmic patterns	Primarily auditory; also tactile	Serves as a temporal anchor, enabling synchronization and the perception of regularity across sensory inputs
*Metre*	The hierarchical organization of beats into groups, often with strong and weak beats	Auditory; occasionally visual	Provides a framework for understanding rhythmic complexity, enabling segmentation and emphasis in patterns

Taken together, the findings reviewed in this section might lead one to conceptualize temporal organization/structure as an amodal structural feature (for a review, see also Aksentijević et al., 2001; Royer & Garner, 1970; Spence & Di Stefano, 2024a).^16^ However, it is unclear whether in those studies what is being picked up by participants is the amodal formal structure of percepts or rather whether these results could be equally explained in terms of a (learned) multisensory integration based on similar/shared perceptual features.^17^ Finally, certain findings (Kang et al., 2018) seemingly hint at the possibility of mapping auditory information to visual and haptic based on the identical temporal profile of the stimuli, thus representing a case of what Spence and Di Stefano (2024b) defined as ‘sensory translation’, based on putatively amodal structural attributes shared between audition, vision and touch (see Spence & Di Stefano, 2024a, Di Stefano & Spence, 2024; we delve deeper into these questions in Sect. "Should rhythm be considered as an amodal stimulus quality?").

From a phenomenological perspective, the recognition of temporal structure in vision or touch does not immediately necessitate the experience of (auditory) phenomena such as rhythm and metre. Furthermore, just because the pattern is presented to the eye or skin surface, say, does not necessarily mean that it will not automatically be converted, or imagined mentally as a sound sequence (see Guttman et al., 2005, on this point). Finally, despite the similarity of temporal grouping processes across modalities (at least their behavioural manifestation), the sensitivity to a ‘beat’ might differ substantially between the auditory and visual/touch modalities, likely due to psychophysical constraints (e.g., see Grahn et al., 2011).

A final remark should be made regarding the nature of touch (or rather the tactile receptor array), which, unlike audition and vision, is spread across the body rather than localized in specific organs such as the ears or eyes. In contrast to vision and audition, the sensitivity of the organ of touch – the skin – is not uniform but varies significantly across different body parts (Weinstein, 1968). Temporal perception in touch is influenced by the density of receptors and the mechanical properties of the skin. For instance, the fingertips, with their high density of Meissner and Merkel receptors, offer superior tactile and temporal sensitivity compared to regions such as the torso or arms, where receptive fields are broader, and receptor density is lower (Ackerley et al., 2014; Gallace & Spence, 2014; Johansson & Vallbo, 1979). These differences suggest that the tactile perception of temporal structure might vary not only by stimulus type but also by the body region stimulated.^18^

## Crossmodal influences of the perception of temporal patterns presented to different senses

### Auditory driving

Given the primacy of experiencing temporal patterns in audition compared to vision and touch, one might ask whether there are crossmodal influences when the temporal patterns are presented to different senses. Numerous studies have demonstrated that auditory flutter dominates the perception of visual flicker (Gebhard & Mowbray, 1959; Knox, 1945a, 1945b; Recanzone, 2003; Shipley, 1964; Wada et al., 2003). When the rate of repetitive auditory stimulus is increased or decreased, while visual flicker remains constant, the latter appears to change accordingly with the auditory stimulus, an effect known as ‘auditory driving’ (Gebhard & Mowbray, 1959). By contrast, changes in the rate of visual flicker do not appear to change the perceived rate of auditory flutter. For instance, Myers and colleagues (1981) had their participants set flicker rate to match flutter, or vice versa. Results showed that the same physical rate of stimulus presentation was perceived differently as a function of the modality.

In a seminal study by Shipley (1964), the participants had to judge the rate at which a sound appeared to flutter or, at other times, to judge the rate at which a light source appeared to flicker. The results revealed that changing the physical rate of flutter of a clicking sound affected the apparent rate at which a flashing light was simultaneously seen to flicker, thus demonstrating that auditory flutter influences the perception of visual flicker.

Welch and colleagues (1986) conducted two studies to investigate crossmodal influences on rate perception. The participants were presented with 4-, 6-, 8- and 10-Hz stimuli in auditory and visual modalities and had to estimate the rate using a magnitude estimation procedure in the following conditions: auditory alone, visual alone, auditory rate perception in the presence of perceptually discrepant visual stimulus, and visual rate perception in the presence of a perceptually discrepant auditory stimulus. The first two conditions are the auditory and visual ‘control’ measures, while the latter provide the ‘bisensory’ measures. The results supported the conclusion that when vision and audition conflict in their information about temporal rate, audition dominates the temporal percept. In a similar study, Recanzone (2003) confirmed that humans discriminate auditory temporal rates better than visual temporal rates. Moreover, the presence of an auditory distractor profoundly influenced the perception of visual temporal rates, while the visual stimuli had no measurable influence on the perception of auditory temporal rates.

Guttman and colleagues (2005) presented three experiments investigating the idea that rhythm, or, in authors’ terminology, the ‘temporal structure’ portrayed solely by visual input receives automatic, obligatory encoding in the auditory domain. The participants in their study were presented with visual sequences of temporally random contrast changes. The participants had to make same/different judgements concerning two sequences of visual stimuli while in the presence of either task-irrelevant auditory or visual distractor temporal patterns. If visually presented temporal structure is encoded auditorily, then incongruent auditory information should presumably impair the processing of the visual stimuli. By contrast, if the comparison of two visual temporal sequences uses visual representations, then incongruent auditory signals should have a minimal effect; however, manipulations that disrupt the visual similarity of the two sequences – even if along a task-irrelevant dimension – should disrupt processing.

Intriguingly, the task-irrelevant auditory distractors interfered with visual judgements, whereas the presence of visual distractors did not. According to Guttman and colleagues (2005), the auditory pattern did not change the observer’s perception of the visual temporal pattern. Instead, observers may have converted or ‘heard’ the visual temporal pattern, often described as rhythm, which could explain why their visual performance was influenced by an incongruent auditory distractor but not by a visual distractor.^19^ Experiment 1 indicated that rhythmic auditory sequences disrupt the processing of visual temporal structure. Experiment 2 further demonstrated that this auditory interference far outweighs the impact of varying the nature of the stimulus changes giving rise to visual temporal structure. Experiment 3 confirmed that crossmodal interference impairs the encoding of the temporal structure, rather than (or in addition to) its retrieval. Together, these findings suggest that the human perceptual system abstracts temporal structure from the nature of its visual ‘messenger’, and may automatically encode this structure in a format that shares key properties with auditory processing, such as rhythmic organization or temporal regularity. That said, subsequent research has vigorously challenged Guttman and colleagues’ controversial claim. In particular, according to the results of research by McAuley and Henry (2010), the auditory encoding of visual rhythms is neither obligatory nor automatic (see also Grahn et al., 2011, for a similar conclusion based on a combined behavioural and neuroimaging study).

Boltz (2017) extended the investigation of the auditory driving effect to cinematic art. The author presented participants with montages (slideshows) of still scenes that had been pre-rated as intermediate in their affective valence and arousal. These were either presented alone or else with music of a similar affect. Most importantly, musical tempo was manipulated such that it was equivalent to or 15% faster or slower than that of the montage. Immediately afterwards, participants were given two visual probes and asked to decide which displayed the same rate as before. One probe was the same while the other was 15% faster or slower. The results revealed that melodies that had tempi that were faster or slower than that of their accompanying visual scenes led participants to falsely recognize these scenes as faster or slower, respectively. The reverse phenomenon, however, did not occur – variations in visual rate did not influence tune rate recognition.

The findings of a subsequent study by Boltz (2018) confirmed that variations in musical tempo biased the perceived rate of visual motion in a corresponding manner, while visual information exerted no influence on auditory rate recognition. The magnitude of this effect was found to depend on the audiovisual affect. When the affect of one sensory modality is congruent with that of the other, it tends to heighten the emotional impact of a scene as well as the perceived rate of visual motion in the presence of auditory tempo discrepancies. Affect incongruency was also reported to give rise to auditory driving but to a lesser extent.

Auditory driving has typically been explained through modality appropriateness (Welch & Warren, 1980, 1986). According to this account, the cognitive system is biased toward perceptual unification, and any discrepancies of a reasonable magnitude are reduced, which may involve one modality dominating the other. The modality that is more appropriate to a particular type of information (e.g., Welch & Warren, 1980, 1986) will typically dominate over the others, assuming that the precision and reliability of information are comparable across the sensory systems (Ernst & Banks, 2002). Given that audition is better at processing temporal information (Repp & Penel, 2004), visual rate will not only be more poorly recognized than auditory rate but biased toward audition (and not vice versa) in the presence of tempo discrepancies – as observed in this section.^20^

### Effects of perceiving temporal patterns on cognitive task performance

Escoffier and colleagues (2010) investigated whether and how a musical rhythm entrains a listener’s temporal attention. Participants were presented with pictures of faces and houses and indicated whether picture orientation was upright or inverted. They performed this task in a silent condition or with a musical rhythm playing in the background. In the latter case, pictures could occur off-beat or on a rhythmically implied, though silent, beat. Both pictures of faces and houses presented without the musical rhythm and off-beat were responded to more slowly than the same pictures presented on-beat. The results suggest that exposure to musical rhythm facilitates concurrent visual processing.

Lagarrigue and colleagues (2021) investigated the effects of auditory stimulation on the procedural learning of a visuo-motor sequence. The experimental procedure included a test of attentional performance and the serial reaction time test. Participants were randomly assigned to one of the following conditions: Visual Only condition; Congruent Audio-Visual condition (an auditory stimulation was presented at the same time as each visual cue); Non-Congruent Audio-Visual condition (an auditory stimulation was presented 200 ms after each visual cue); Regular Rhythmic Auditory Stimulations condition (auditory stimulations were presented every 500 ms independently of visual stimulations and participants’ responses); Irregular Rhythmic Auditory Stimulations condition (auditory stimulations were presented irregularly and independently of visual stimulations and participants’ responses); Quick Regular Rhythmic Auditory Stimulations condition (auditory stimulations were presented every 300 ms independently of visual stimulation and participants’ responses). The results suggest that both congruent audio-visual stimulation and regular rhythmic auditory stimulation promote procedural perceptual-motor learning, while auditory stimulation with an irregular (or very quick) tempo hinder learning.^21^

Zhao and colleagues (2024) investigated perceptual learning of crossmodal (auditory-visual or visual-auditory) temporal interval discrimination (TID) and its impacts on unimodal (visual or auditory) TID performance. In their experiments, participants had to indicate whether the first or second pair of stimuli had a longer interval. The results revealed that learning to discriminate a 200-ms crossmodal temporal interval, defined by a pair of stimuli, one visual and the other auditory, enhances unimodal visual and auditory TID performance. Moreover, the crossmodal TID training also minimized unimodal TID thresholds with a pair of visual or auditory stimuli at the same interval, even if crossmodal TID thresholds are multiple times higher than unimodal TID thresholds. Subsequent training on unimodal TID failed to reduce unimodal TID thresholds further. These results indicate that learning of high-threshold crossmodal TID tasks can benefit low-threshold unimodal temporal processing, which may be achieved through training-induced improvement of a conceptual representation of sub-second timing in the brain.

Crossmodal congruency effects based on stimulus identity (in this case defined by a simple temporal pattern) have been documented in several studies (Frings & Spence, 2010; Mast et al., 2014). For example, Frings and Spence presented two rhythms to participants’ eyes, ears and/or hands in a four-alternative rhythm discrimination task. Stimulus identity and stimulus modality were varied orthogonally. When the target and distractor rhythms were presented in different senses, significant crossmodal congruency effects were observed in all conditions (i.e., performance on the incongruent distractor trials was significantly more error-prone than on the congruent distractor trials). These crossmodal distractor effects were based on the identity of the target rhythm. Intriguingly, the magnitude of the crossmodal congruency effects differed as a function of the target modality, but were unaffected by the modality of the distractor.

### Interim summary

The literature published so far clearly demonstrates that audition dominates over the other senses in terms of sensitivity to alteration in the temporal pattern, and in terms of modifying the perceived temporal structure of sequences presented in the other senses when presented simultaneously (and regardless of whether the physically same temporal pattern is presented in the different senses or not; e.g., Goodfellow, 1934; Grahn et al., 2011; Grondin & McAulely, 2009; Kolers & Brewster, 1985; Llamon & Goldstone, 1974; Recanzone, 2003, 2009; though see Guttman et al., 2005).^22^ Typically, tactile performance in temporal tasks, whether of the perception or production (of rhythm) type, tends to fall in-between that of auditory and visual performance (e.g., see Gault & Goodfellow, 1938; Kolers & Brewster, 1985), with the beat being successfully and easily extracted from sequences of tactile stimuli as well (Brochard et al., 2008). Furthermore, differences in tempi are shown to affect higher cognitive tasks (e.g., those involving visual attention and procedural/perceptual learning).

**Fig. 2:**
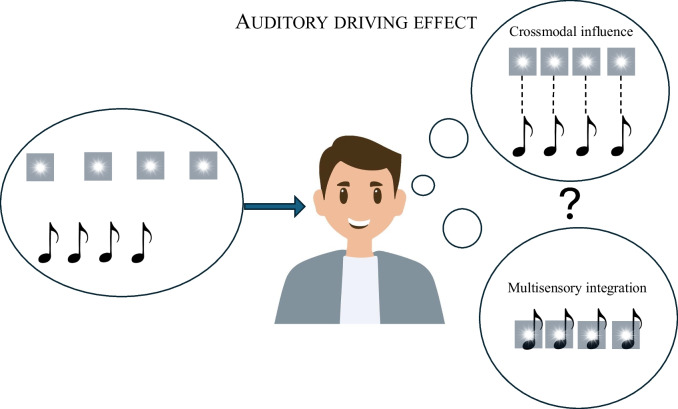
The auditory driving effect in the audiovisual domain could be conceptualized as resulting from the crossmodal influence over audition on vision or from multisensory integration of individual unisensory inputs, audiovisual. Human figure adapted from content freely available on Vecteezy.com

One question that remains open, at least in part, concerns whether the results of studies on auditory driving effect should be explained in terms of the crossmodal influence of one sense on another, or as a form of multisensory integration where several unisensory inputs are combined into a unified perceptual Gestalt, or both accounts are legitimate (see Fig. 2). Solving this issue may depend on whether the ensuing perceptual organization is experienced as unitary (a form of Gestalt crossmodal grouping) or as unimodal patterns that are similar (a kind of perceptual similarity or crossmodal correspondence-based explanation).

## On the perception of temporal structure in crossmodally presented patterns

We now move to the crossmodal perception of temporal structure, that is, when some proportion of the temporal information is presented only to a single sensory modality and the rest to the other modality(ies). The latter can be related to what in the field of psychology have been defined as ‘inter-sensory Gestalten’ (Gilbert, 1938) or ‘transmodal Gestalt’ (Kubovy & van Valkenburg, 2001), concepts seemingly implying that people can extract structures from multisensory elements.^23^ The key question here regards the possibility of perceiving an emergent temporal organization that is not present or perceptible in any of the individual sequences of unisensory stimuli. This question is significant not only within psychology but also in broader fields, as Grahn and colleagues (2011, p. 1231) noted: “How we measure time and integrate temporal cues from different sensory modalities are fundamental questions in neuroscience”. Surprisingly, however, despite the importance of this question, there is a striking lack of empirical research on the topic, with the few studies trying to shed light on this question failing to provide convincing evidence for intersensory, or transmodal, Gestalts (see Spence & Di Stefano, 2022, on the idea of crossmodal harmony).

The previously cited studies by Handel and Buffardi (1968) and Jokiniemi et al. (2008) also involved a crossmodal condition. In Handel and Buffardi’s study, the participants had to identify temporal patterns of a sequence of elements presented in different modalities. The participants were exposed to the stimulus until they could identify the pattern successfully. Besides confirming that temporal patterns presented in the auditory and tactile modalities were easier to identify than those presented in the visual modality (see Sect. "Comparing the perception of temporal structures in sequential unimodal auditory, visual and tactile stimuli"), the results showed that participants perceived crossmodal patterns as structured (see also Handel & Buffardi, 1969). In Jokiniemi and colleagues’ (2008) study, the rhythmic patterns emerged from the combinations of two sensory modalities (i.e., auditory-tactile, tactile-auditory, auditory-visual, visual-auditory, tactile-visual, and visual-tactile). The results of a same-different discrimination task showed that the auditory-tactile had the highest rate of correct responses (75%), while all other combinations had less than 70%, the least accurate being the visuo-tactile combination (see also Rubinstein & Gruenberg, 1971).

The pioneering study by Huang and colleagues (2012) provided some of the only evidence published to date that people can efficiently extract the musical metre from a temporal sequence of elements, some of which happen to be presented auditorily, others via the sense of touch. Participants had to discriminate between two types of temporal sequences, ‘duple’ (march-like rhythms) and ‘triple’ (waltz-like rhythms) that were presented in three conditions, namely unimodal (auditory or tactile alone), bimodal (where sequences were distributed between the auditory and tactile channels such that a single channel did not produce coherent metre percepts), and simultaneously presented bimodal inputs where the two channels contained congruent or incongruent metre cues. The results revealed that metre is perceived similarly well (70–85%) in the unimodal condition, independently of the sense modality being stimulated. In the bimodal experiments, when auditory and tactile cues are integrated to produce coherent metre percepts, performance is high (70–90%) when all of the metrically important notes are assigned to one channel only, and is reduced to 60% when half of these notes are assigned to one channel. When the important notes are presented simultaneously to both channels, congruent cues enhance metre recognition (90%).

The results of Huang and colleagues’ (2012) study suggest that the mechanisms underlying tactile and auditory metre perception share similar characteristics. Moreover, the bimodal task indicates that auditory and tactile inputs are grouped during metre perception. Noteworthily, the participants performed all of the experiments without training, feedback or instructions about where to focus their attention, thus demonstrating that auditory-tactile integration for metre perception is an automatic process. These results might therefore be taken as providing support for the claim that audiotactile musical metre perception constitutes one of the first genuinely intersensory Gestalten to have been documented to date. However, one should be careful before drawing such a conclusion, given the long history of research into crossmodal perceptual organization, where perceptual switching appears to occur independently in different senses, and where despite many attempts to demonstrate/find it, there is little evidence of genuinely crossmodal apparent motion, say (for reviews, see Spence, 2015; Spence et al., 2007). Despite the existence of parallel and similar organizational principles at play in the various senses (see Huddleston et al., 2008; Lakatos & Shepherd, 1997; Spence et al., 2007), and despite plenty of evidence of crossmodal influences on perception, perceptual grouping that genuinely spans across multiple senses remains, then, noticeable by its absence^24^ (see Table 3 for a summary of the main findings).

**Table 3:** Main findings. Table summarizing the key findings from the literature on the perception of temporal structure(s) within and across the senses, organized by sensory modality, mode of presentation (unimodal vs. crossmodal), and the temporal structure involved (beat, metre, pattern)

	Sense	Temporal structure			Source(s)
		Beat	Metre	Pattern	
Unimodal	Audition	Yes	Yes	Yes	Bharucha & Pryor, 1986 Brochard et al., 2008 Chang & Trehub, 1977 Grahn et al., 2011 Grondin & McAuley, 2009 Handel & Buffardi, 1968, 1969 Huang et al., 2012 McAuley & Henry, 2010 Marks, 1987 Kang et al., 2018 Repp, 2005
	Vision	Yes	Yes	Yes	Grahn et al., 2011 Grondin & McAuley, 2009 Handel & Buffardi, 1968, 1969 Marks, 1987 McAuley & Henry, 2010 Kang et al., 2018
	Touch	Yes	Yes	Yes	Brochard et al., 2008 Handel & Buffardi, 1968, 1969 Huang et al., 2012 Marks, 1987 Kang et al., 2018
	Olfaction	No	No	No	N/A
	Gustation	No	No	Yes/?	Von Békésy, 1964
Crossmodal	Audiovisual	Yes	Yes	Yes	Collier & Logan, 2000 Grondin & McAuley, 2009 Guttman et al., 2005 Handel & Buffardi, 1968, 1969
	Audiotactile	Yes	Yes	Yes	Handel & Buffardi, 1968, 1969 Huang et al., 2012
	Visuotactile	Yes/?	Yes/?	Yes	Handel & Buffardi, 1968, 1969

Notably, in the “crossmodal” studies, only the study by Huang and colleagues provided participants with a temporal pattern that emerged from the combination of stimuli from different senses. All of the other studies included in the “crossmodal” section of this table compared the perception of temporal patterns across different sensory modalities presented sequentially (with the exception of Handel & Buffardi, 1969, who used the simultaneous presentation of stimuli from different modalities but for a different purpose/ experimental task). When the empirical results appear to support the perception of temporal structure but do so in an inconclusive manner (e.g., due to the indirect investigation of the notion of rhythm), we have chosen to denote this with ‘Yes/?

## Discussion

After reviewing the literature on the perception of temporal patterns within and across the senses, several key questions emerge that remain partially unanswered, yet are crucial for advancing our understanding of this complex phenomenon. In the following discussion, we address these questions, which span topics in sensory psychology, including amodality, synchrony, multisensory integration, and the potential biological advantages associated with the ability to perceive temporal organization in multisensory or crossmodal contexts.

### Is it possible to perceive the temporal structure of stimuli in the chemical senses?

Going back to one of the key questions that inspired this review, we might conclude that, with the possible exception of von Békésy (1964),^25^ the ability to perceive temporal structure appears to be largely confined to the so-called spatial senses: audition, vision and touch. Such an answer raises the related question of why temporal patterns, such as rhythm and metre, are not perceived in the chemical senses (e.g., olfaction and gustation).^26^ In what follows, we examine some of the psychophysiological constraints characterizing chemosensory perception that might prevent humans from perceiving temporal structure in these domains.

First, the physical properties of chemical stimuli (odours and tastes) differ significantly from those of mechanical and electromagnetic stimuli (sounds, light and tactile sensations). Chemical stimuli often rely on diffusion or transport through a medium, such as air or saliva, which inherently introduces variability and delays. This variability disrupts the regularity needed to establish a temporal pattern, making it difficult, if not impossible, for the sensory system to perceive rhythm or metre. However, it is worth noting that gustatory information is temporally coded. Different tastants can evoke neural responses of equal magnitude but with distinct temporal firing patterns, both across neurons and within individual neurons (Mistretta, 1972; for a review, see Hallock & Di Lorenzo, 2006). Despite this, such low-level coding does not seem to have any direct implications for the ability to perceive temporal structure in a sequence of gustatory stimuli.

Second, the temporal dynamics of the chemical senses are much slower as compared to the rapid processing required for rhythm perception.^27^ For audition, vision and touch, a conservative estimate for the minimum values of stimulus duration and interstimulus interval (ISI) for people to perceive subsequently presented stimuli as separated can be of 100 ms and 100 ms, respectively. These values are lower than most values we can find in the empirical literature for olfaction and taste (Schriever et al., 2015, for a review; though see von Békésy, 1964, for a possible exception).^28^ Olfactory and gustatory receptors have slower transduction latencies, and the processing of these chemosensory stimuli typically involves slower transduction latencies of information.^29^ In addition to the speed of transduction, the properties of the stimuli may also play a role. Most auditory inputs that evoke beat perception feature sharp transients (i.e., sudden onsets and offsets), whereas the onset and dissipation of tastes and odours seem to occur more gradually, thus making these stimuli less suitable for rapid sequential presentation than clicks or lights.^30^

Furthermore, we might speculate that the evolutionary role and utility of the chemical senses may not align with the need to perceive temporal patterns (see Sect. "Are there biological advantages in perceiving temporal organization?"). Auditory, visual and tactile rhythms often play a critical role in movement control, communication, and social interaction – domains in which timing is crucial. In contrast, the chemical senses are more closely associated with detecting and discriminating the presence of specific substances, such as food, toxins or pheromones, where the precise timing of stimulus delivery is less critical.

Significant differences between the spatial and chemical senses arise in how odours and tastes are experienced. Tasting and smelling typically involve intermittent, active sampling of the chemosensory environment. In olfaction, respiratory rhythms and sniffing rates provide the basic pattern for intermittent odorant input (see Laing, 1983, 1985, 1986; Laing & MacLeod, 1992; see also Olofsson et al., 2013), while in gustation, ingestive acts are the primary source of stimulation (for a review, see Halpern, 1983). The active nature of these actions may limit the likelihood of perceiving temporal structure in the stimuli since the temporal structure is likely determined by the active sensing (sniffing) rather than by the temporal properties of stimulus itself (though see Wilson, 2023).

A caveat should be noted regarding the perception of temporal order in gustation (taste). While there is a significant body of literature emphasizing the importance of properly sequencing flavour experiences – particularly in how dishes or wines are presented to attendees – these studies primarily focus on the impact of temporal order in the presentation of foods and drinks (e.g., Spence et al., 2017; Wang et al., 2019). One might consider the temporally evolving experience of wine tasting, where the process begins with smelling the bouquet or aroma, followed by tasting the wine through its (temporally) distinct phases: The initial attack, the mid-palate, and the finish. However, what is missing in this context, as compared to the concept of rhythm discussed in this review, is the repetition of identifiable stimuli, such as beats, which can create a temporal structure. In gustation, the focus is on the sequence in which items are presented, rather than on the emergence of a structure/pattern based on repetition of elements.^31^

That being said, one might argue that what qualifies as rhythmic in olfaction (or gustation) is debatable, partly due to the absence of a clear and universally accepted definition of rhythm. Expanding the perspective beyond the musical context to encompass broader temporal aspects of perception, one could adopt definitions of rhythm that might also include olfactory events (see also Spence, 2021, on the intriguing notion of “scented sounds”). For instance, Cooke and Myin (2011) proposed the concept of ‘olfactory trills’,^32^ which might involve alternating perfumery strips with two different odorants presented to the nose. While such an idea preserves the fundamental alternation of ‘events’ (whether identical or contrasting) that typically characterizes rhythmic structures, it may lack certain features that are crucial for perceiving a strong sense of rhythm. These features might include sharp onset and offset dynamics or slower temporal variations, both of which are often essential for the experience of rhythm in other sensory modalities.

### Should rhythm be considered as an amodal stimulus quality?

One assumption that is seemingly implicit in the reviewed literature is the idea that the principles of perceptual organization operate in an equivalent manner along the same stimulus dimensions. For instance, commenting on his findings on the similarity of judgements of temporal patterns across the senses, Marks (1987, pp. 255–256) observed: “The fact that individual subjects are consistent across modalities in their use of these dimensions suggests a supramodal strategy for evaluating, judging, and comparing these temporal patterns […] it seems clear that people can and do employ a general, amodal strategy or strategies – perhaps parallel to modality-specific mechanisms – to judge temporal patterns quite independently of their sensory modality of origin”. While, at first sight, much of the reviewed evidence seemingly supports  Marks’ observation, the evidence that temporal patterns can easily be matched, or recognized, across the senses does not necessitate the subjective experience of rhythm, nor does it necessitate, that it is the same quality/property that is being perceived across modalities nor that we are looking at a structural feature that is ‘amodal’ (for a review, see Spence & Di Stefano, 2024a).

Similar findings might be well explained in terms of analogical mapping, albeit with the same Gestalt rules/principles operating in parallel and independently (for a review, see Spence, 2015) in each of the senses. One might therefore admit the perception of similar structures in different senses without necessarily having to use the problematic term amodal (see Spence et al., 2013), rather appealing to use the terminology of ‘analogous structure’, ‘analogous grouping principles’, or ‘structural isomorphisms’ between the senses (see Spence & Di Stefano, 2024a, 2024b). However, such a pathway is not free from issues, being connected to the more fundamental, and intricate, question of whether it is even possible to experience similarity between stimuli presented to different sensory modalities: analogical mapping should by no means imply the production of analogical phenomena (see Di Stefano & Spence, 2024).

Moreover, while the concept of an amodal temporal processor may seem intuitively appealing, several questions emerge regarding its precise workings, particularly its purported amodal nature. For example, how should we interpret findings that support the auditory driving effect? Why does the so-called amodal temporal processor appear to function differently across the senses, for example, by privileging auditory inputs both in unimodal and crossmodal domains (e.g., Jokiniemi et al., 2008)? Does this asymmetry reflect a hardwired neural mechanism, or does it emerge from Bayes-optimal integration based on the precision of sensory inputs? Relatedly, to what extent can variations in temporal processing across modalities still be considered expressions of the same amodal processor? Furthermore, does the evidence indicating that auditory temporal resolution surpasses visual temporal resolution fundamentally challenge the notion of such an amodal temporal processor?

The phenomenon of crossmodal mental imagery may also be relevant in this context. Defined as the spontaneous formation of a mental image in one modality when a stimulus is physically presented (or imagined) in a different modality (for a review, see Spence & Deroy, 2013), crossmodal mental imagery could potentially explain the perception of auditory-like temporal features (e.g., beat, metre) in stimuli presented in vision and touch. In those occurrences, being exposed to the rapid presentation of tactile or visual stimuli might evoke the image of an auditory rhythm which therefore could mediate for the perception of the temporal structure in audition and vision.

### Are there biological advantages in perceiving temporal organization?

Given the widespread ability to perceive temporal structuring in audition and beyond, one might ask whether such an ability provides any biological advantages that extend beyond mere perceptual processes. First and foremost, as observed by Liebermann (1973), the ability of the vocal tract and brain to produce and perceive rapidly changing sound signals can be seen as a physiological prerequisite for the emergence of language in evolutionary biology. As pre-humans evolved, the ability of the vocal tract and brain to deal with fast changes and timing differences improved as linguistic ability became increasingly important for survival and reproduction (cf. Parncutt, 2024, p. 103).

A growing body of comparative research suggests that the ability to perceive and synchronize to temporal patterns may have evolved not just for individual survival but also to enhance social cohesion and group dynamics. The capacity for perceiving temporal structure has been closely linked to the evolution of vocal learning and rhythmic pattern perception, which are crucial for communication in many species (Patel, 2021). Studies on vocal learning species, such as songbirds, have shown that these animals can recognize rhythmic patterns and adapt to different temporal intervals, a skill that appears to be less developed in non-vocal learners (Rouse et al., 2021). In songbirds, rhythmic pattern perception is not just a byproduct of auditory processing but is essential for mating calls and territorial displays, which are key to social interactions and reproduction (Rouse et al., 2021). Moreover, the neural circuitry involved in vocal learning and rhythm perception in these species shows significant overlap with the regions involved in social bonding and communication (Patel, 2021; Rouse et al., 2021).

The ability to perceive and synchronize with temporal patterns has significant biological implications also for humans, particularly in the realm of social bonding. Synchrony, the alignment of actions in time between individuals, is a powerful social glue that can enhance group cohesion and cooperation. This is evident in activities such as group singing, dancing and marching, where synchronized movements create a sense of unity and shared purpose (Woolhouse, 2023).

Synchronous movement between adults has been shown to increase group cohesion, social cooperation, trust and affiliation between those involved, even among strangers (for a review, see Trainor & Cirelli, 2015). Furthermore, people are more likely to engage in altruistic acts (defined as acts that require personal sacrifice) aimed at people with whom they previously moved in synchrony compared to out of synchrony. This is already evident in 4-year-old children, with joint synchronized movement (i.e., swinging) influencing cooperative behaviour among peers, by decreasing the time required for completing two joint tasks, thus indicating better cooperation between the children (Rabinowitch & Meltzoff, 2017; see also Rabinowitch & Knafo-Noam, 2015). Overall, the reviewed studies strongly support the idea that the ability to perceive the temporal structure of external stimuli and to synchronize with beats is one of the elements that favoured the development of complex social structures, fostering prosocial actions and enabling individuals to coordinate their actions and communicate effectively within a group.

That being said, one could observe that the evolutionary benefits of perceiving temporal structure – such as social bonding – seem primarily linked to the perception of auditory stimuli. Humans’ ability to synchronize with external stimuli is exceptional when those stimuli are auditory. One could speculate that the evolutionary root of the perception of temporal structure lies in audition or, at most, the audiovisual domain. Vision, in this context, might serve as an additional means of accessing the same information, such as when we see people clapping instead of hearing the sound.

Tactile perception might also contribute, but its role in perceiving temporal rhythms is less common in everyday life and is primarily studied in laboratory settings. This leads to the idea that humans evolved primarily to perceive auditory rhythms, and that the ability to perceive visual or tactile rhythms could be viewed as cognitive byproducts – or “spandrels” – of this primary auditory ability (Gould & Lewontin, 1979).

**Fig. 3:**
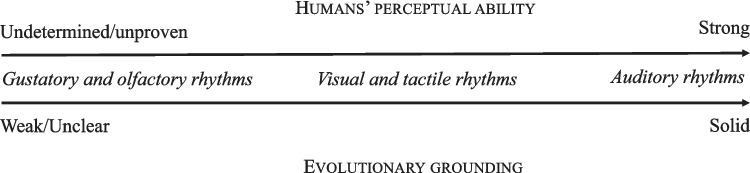
Conceptual representation comparing the perception of temporal structure across sensory modalities. The spatial senses (vision, audition and touch) exhibit a strong capacity for perceiving temporal structure, which has significant biological relevance for key activities like communication and socialization. In contrast, the perception of temporal structure in the chemical senses (taste and smell) is either absent or yet to be tested, with no clear biological role identified for humans

This reasoning further supports the idea that perceiving temporal structure is not an amodal quality, but rather an ability rooted in audition. While it can occasionally be abstracted and extended to the spatial senses, it is unlikely to be extended to the chemical senses, given the inherent limitations of those modalities, at least in the way it is experienced in audition or vision (see Fig. 3).

Finally, the intersensory redundancy hypothesis (IRH) should also be mentioned here. The IRH posits that during early development, infants are particularly sensitive to information that is redundantly presented across multiple sensory modalities. This redundancy, particularly in temporal information, is thought to facilitate the integration of sensory inputs, enhancing perceptual learning and cognitive development. According to the IRH, temporal features such as rhythm, synchrony and tempo, when simultaneously presented through different senses – such as auditory and visual – become more salient to infants, helping them to better detect and process these features.

The relevance of the IRH for the perception of temporal structures across the senses lies in its ability to explain how humans, from an early age, develop the capacity to perceive and integrate temporal patterns. For instance, when an infant hears a rhythmically regular sound (e.g., a drumbeat) and simultaneously sees a visual stimulus (e.g., a light flashing in sync with the sound), the redundant temporal information across these modalities is more easily perceived and learned. This facilitates the development of a unified, multisensory perception of temporal structures, where the brain can effectively integrate and synchronize inputs from different senses to create a cohesive experience.

### Temporal correlation, synchrony and multisensory integration

One additional question that underlies the research reviewed here concerns the conditions that enable perceivers to integrate temporal information received from different senses (which is, in turn, a crucial condition to perceive crossmodal rhythm). While we will not delve deeply into the broader topic of multisensory integration, it is important to highlight a couple of key points that are relevant to our discussion. Kubovy and Schulz (2010) argue that binding between auditory and visual stimuli occurs only when the component stimuli are presented simultaneously and when there is a plausible common cause. This concept, often referred to as a coupling prior in Bayesian decision theory (for a review, see Chen & Spence, 2017), suggests that for effective integration to occur, the brain must infer a shared origin or cause for the stimuli. Schutz and Kubovy’s (2009) work on causality and crossmodal integration supports this idea, emphasizing that simultaneous presentation alone is not sufficient without an inferred common cause.

There is also a distinct body of literature focusing on the temporal correlation of signals as a cue for multisensory integration (Parise et al., 2012, 2013). Building on earlier work by Radeau and Bertelson (1987), these studies demonstrate that temporally correlated auditory and visual pulse trains are more likely to be bound together. Parise and colleagues (2012) further explored this by examining the role of temporal correlation between auditory and visual signals in causal inference. In a localization task involving visual, auditory and combined audiovisual targets, they found that participants’ precision improved optimally in combined conditions only when the audiovisual signals were correlated. This finding implies that similarity in the temporal structure of multisensory signals is essential for humans to move from recognizing mere correlation to inferring causation, thereby strengthening sensory integration.

Taken together, the effects of temporal correlation highlighted here should be viewed as part of the broader influence of temporal structure on sensory integration, both within and across modalities. Temporal correlation may indeed represent the key physical property underlying the Gestalt law of grouping by ‘common fate,’ where similar temporal patterns lead to the perception of a unified sensory experience.

## Conclusions

The evidence summarized in this paper clearly demonstrates that the perception of temporal patterns, primarily conceived as an auditory phenomenon, extends well beyond audition into the realms of vision and touch. This multisensory capability suggests that temporal organization is a fundamental aspect of human perception, potentially underpinned by shared neural mechanisms across sensory modalities.

The crossmodal influences observed, particularly the dominance of auditory stimuli in shaping the temporal structure perceived in other senses, highlight the intricacies of multisensory integration. The results from a couple of studies (Handel & Buffardi, 1968; Huang et al., 2012) suggest that the mechanisms underlying tactile and auditory metre perception share similar characteristics, thus providing support for the claim that audiotactile musical metre perception constitutes one of the first genuinely intersensory Gestalten to have been documented to date. However, the rarity of such phenomena, especially within the spatial senses, aligns with the idea that intramodal grouping processes typically take precedence over crossmodal organization, especially when all the relevant stimuli are provided within the same modality (Spence & Chen, 2012). Moreover, the crossmodal influence of temporal patterns may not only depend on auditory dominance but also on tactile variability, with audio-tactile integration likely being modulated by the regional sensitivity of the skin surface stimulated (e.g., fingertips vs. back), thus suggesting a more nuanced interaction between the senses.

The exploration of whether temporal patterns can be perceived across different modalities raises important questions about the existence of an amodal or supramodal temporal processor in the brain (for a review, see Spence & Di Stefano, 2024a, 2024b). The findings suggest that while temporal patterns can be recognized across modalities, the mechanisms involved may not necessarily be amodal but rather reflect a learned integration of similar perceptual features across senses.

Finally, evolutionary and comparative research has highlighted that the ability to perceive and synchronize with temporal structures may not simply be a passive sensory process but rather an evolved mechanism to strengthen social bonds and enhance group survival (e.g., Carouso-Peck et al., 2021). While this ability has been widely demonstrated in the spatial senses, its existence and biological significance in the chemical senses remain unexplored (see Fig. 3).

### Open questions and future directions

The reviewed literature raises several research questions that could be empirically addressed in the future to shed light on aspects of temporal structure perception that currently remain unclear. Here, we consider five open issues/potential directions:

1) First and foremost, given the scarcity, if not complete absence, of experiments focusing on the perception of temporal structure in the chemical senses, intriguing research could involve developing empirical protocols that implement rhythmic perception tasks using odours or tastes/flavours. These protocols should specifically assess the ability to perceive temporal features (e.g., beats) within a sequence of trigeminal olfactory or gustatory stimuli, while ruling out participants’ ability to identify the quality or nature of the stimuli (e.g., the specific odorant used). This would require optimizing the experimental apparatus to accommodate the unique characteristics of these senses. Such findings could clarify whether the current limitations of research to the spatial senses are due to biological constraints or technical challenges in designing protocols suitable for testing with chemical senses. Addressing either of these possibilities would represent a significant step forward in understanding the multisensory perception of temporal structure. Interestingly, if protocols implementing rhythmic gustatory or olfactory stimuli yield positive results, further research could explore cross-modal designs that combine different senses, including the chemical senses, to examine mutual influences. For instance, researchers could investigate whether the auditory driving effect also impacts the chemical senses. In contrast, if these protocols reveal that observers do not perceive temporal structure in sequences of stimuli presented to the chemical senses, one could still explore whether perceivers can entrain to an auditory beat through odours and tastes.

2) Perceptual and discrimination experiments have been conducted primarily (if not only) in unimodal (e.g., auditory, visual, tactile) or bimodal conditions (e.g., auditory-visual or auditory-tactile). While trimodal conditions involving auditory, visual and haptic modalities have been used once in a sensorimotor synchronization task (Johnson et al., 2020), they have never been applied to test the perception of crossmodal rhythms emerging from the combination of stimuli across all three modalities. Such a protocol could provide valuable insights into the longstanding issue of intersensory Gestalten, or crossmodal Gestalts as they are also known. Additionally, varying the amount of information conveyed by each modality could enhance our understanding of the specific contributions each modality makes to the perception of temporal structures.

3) When considering the temporal variables that influence the integration and perception of crossmodal rhythms, it is important to delve deeper into the relationship between temporal structure and its constituent elements. A key question that arises is the minimum number of elements required for humans to perceive a temporal structure. While very simple patterns may be recognized with only a few elements, more complex structures might necessitate a greater number of elements and multiple cycles before they can be accurately perceived.

Additionally, it is worth exploring if the perception of temporal structure – whether within a single sense or across multiple senses – breaks down when the presentation of the pattern is slowed beyond a certain threshold. This raises the question of whether there is a critical tempo below which temporal structure is no longer perceived as cohesive. Empirical studies could be designed to address these questions, offering a more precise understanding of the temporal thresholds related to the perception of temporal patterns across the senses. Such research would refine our knowledge of how temporal structure is processed and integrated, providing valuable insights into multisensory perception.

4) Further research in the field of artistic practices could explore the intersection of haptics technology and music, as exemplified by Gunther and O’Modhrain (2003) (for reviews, see Papetti & Saitis, 2018; Volta & Di Stefano, 2024). They introduce the concept of tactile composition, or aesthetic composition for the sense of touch, through a system designed to facilitate the creation and perception of complex, musically-structured spatiotemporal patterns of vibration on the body surface. Meanwhile, in 2015, Klissouras created a haptic installation named *Skin Air | Air Skin*, which consists of a rhythmical exploration of air pressure on the skin. The same technology has been used for the work *Air of Rhythms—Hand Series*, created in collaboration with Alexandros Kontogeorgakopoulos (see Kontogeorgakopoulos, 2023).

These innovative approaches are grounded in the fundamental similarities between the senses of hearing and touch, particularly in their shared ability to perceive and process vibrations. Research in the psychophysics of touch has shown that the perceptual ranges and discriminatory limits of these two senses are, in some respects, compatible and overlapping. This suggests that the skin may be capable of processing and appreciating tactile compositions in a manner analogous to how the ear processes music, using parameters such as rhythm, frequency, intensity and duration (e.g., Branje et al., 2010).

Building on this concept, Gunther and O’Modhrain (2002) propose the idea of a ‘crossmodal counterpoint’, where different sensory stimuli are combined to create a multisensory experience. This approach embodies the longstanding idea of translating sensory experiences across modalities, as discussed by Spence and Di Stefano (2024b). However, a challenge remains in ensuring that humans can meaningfully understand the relationship or translation between tactile and auditory stimuli, especially when these are based on psychophysical criteria or allegedly amodal features. These issues are also crucial for developing devices that enable people with hearing impairments to experience musical features through haptic feedback.

An alternative avenue for crossmodal translation could involve leveraging the shared emotional meanings of stimuli. Research has shown that emotional congruence can mediate crossmodal correspondences, particularly in the audiovisual domain (e.g., Di Stefano et al., 2024). This approach might offer a more intuitive and accessible way to explore the connections between different sensory modalities in artistic practices.

5) Findings on the crossmodal influences or association involving rhythms might be potentially relevant for the design of tactons, or tactile icons, conceived as structured patterns of tactile stimuli designed to convey information through touch. Tactons use variations in rhythm, intensity and duration to communicate different messages (e.g., see Brown et al., 2005, 2006). In human–computer interaction, tactons serve as a tactile language, providing rhythmic feedback that users can perceive even when visual or auditory cues are unavailable or impractical.

The rhythm of a tacton is carefully crafted to convey specific meanings: a steady, pulsing rhythm might signal a notification, while a rapid, intense sequence could indicate urgency. These rhythmic patterns are essential in wearable technology, mobile devices and assistive tools for those with sensory impairments, offering a silent yet powerful means of communication. The success of tactons hinges on the human ability to discern and interpret these rhythmic tactile patterns, making them a crucial element in the design of multisensory interfaces (that is vibrotactile icons; see Gallace & Spence, 2014). Furthermore, future studies in this direction should consider not only the characteristics of rhythms but also the specific sensitivity (e.g., spatial/temporal acuity) of the skin in the specific body regions that are stimulated.

Taken together, the above sketched research efforts will eventually shed light on an underlying crucial theoretical question, which currently remains at least partially unanswered. Do we perceive temporal structure directly, or we perceive specific elements – such as rhythm, beat or metre – that embody and represent temporal structure? This leads to a deeper question: Do we perceive temporal structure itself, or do we first perceive something else that makes this structure recognizable?

The conditions necessary for perceiving temporal patterns are fundamental to this discussion. Before rhythmical patterns can emerge, there must be a mechanism that binds the elements of these patterns together. But what exactly provides this ‘glue’ that allows us to perceive temporal structure? Is it rooted in low-level perceptual processes, such as causality or synchrony, where stimuli are automatically bound together by their temporal correlations? Or is it governed by higher-level attentional and cognitive mechanisms that organize and interpret these patterns?

The directions outlined in this review aim to address these questions by investigating the conditions under which temporal structure becomes perceivable. By probing the roles of perceptual, cognitive and attentional mechanisms, future research can clarify whether temporal structure is an inherent perceptual experience or a construct that emerges from more fundamental processes. Ultimately, this inquiry not only deepens our understanding of how we perceive time but also informs broader theories of multisensory integration and crossmodal perception.

## Data Availability

No new data were created or analysed in this study.

## Footnotes

1 This review does not address the emergence of those formal structures, such as fugue, sonata, or rondo, that are the object of music analysis. Perceiving these structures likely requires some theoretical knowledge, in addition to a well-trained musical memory (e.g., Temperley, 2004). Moreover, recognizing them typically requires participants to listen for dozens of seconds, if not minutes, depending on the specific composition.

2 However, as shown by the early findings reported by Warren and colleagues (1969), the perception of separate auditory events as discrete does not always necessarily lead to the identification of the temporal sequence in which they occurred.

3 One might also consider crowding, a phenomenon whereby the perception of a target stimulus is impaired or altered due to the presence of other stimuli presented close in time and/or space (e.g., Vatakis & Spence, 2006). To mitigate this effect, most experimental designs testing perceptual abilities deliberately minimize the number of non-target stimuli presented or sensory modalities stimulated.

4 Tactile temporal perception is primarily mediated by specific mechanoreceptors, including RA1 (Rapidly-Adapting Type 1) receptors and Pacinian corpuscles. RA1 receptors are sensitive to low-frequency vibrations and are involved in detecting tactile onsets and fine textures, while Pacinian corpuscles, which respond to higher-frequency vibrations, play a key role in perceiving fast vibrations and temporal events. These receptors are crucial for detecting changes in vibration and rhythmic patterns, making them essential for tactile perception of temporal structures.

5 The waveform of an acoustic speech signal reveals a sequence of increments and decrements in signal amplitude. Commonly referred to as the speech envelope, this modulation has been shown to exhibit compelling temporal regularity affecting speech comprehension. Studies revealed an increase in power for frequencies between 2 and 8 kHz, with a notable peak between 4 and 5 kHz. Critically, these features are preserved across speakers, languages and speaking conditions (e.g., interviews, telephone conversations, or audiobooks; Poeppel & Assaneo, 2020).

6 Studies demonstrate that the notion of regularity should not be conceived too rigidly. For example, we tend to hear as regular sequences that are not, and small variations are typically ignored in order to preserve patterns of regularities (e.g., von Ehrenfels, 1988). Such a principle applies to both melodic and rhythmic information. Thus, if a sequence of clicks is slightly irregular but all the intervals remain within the window of tolerance, then our perception ‘adjusts’ the irregularities, and we perceive the sequence as the succession of the ‘same’ temporal intervals (Drake & Bertrand, 2003). Similarly, a melody tuned at 440 Hz and at 443 Hz will be likely recognized as the *same* melody.

7 Chapin et al. (2010) showed that selective attention to rhythms led to increased activation in basal ganglia, and basal ganglia activity was observed only after the rhythms had cycled enough times for a stable pulse percept to develop.

8 Somewhat surprisingly, although the use of interval ratios is common when talking about consonance and dissonance, only a few researchers talk in terms of consonant and dissonant rhythms, and such usage is primarily limited to a few musicologists. The terms ‘rhythmic consonance’ and ‘rhythmic dissonance’ do, for example, appear in Schillinger’s System of Musical Composition (1946) where he refers to such combinations as ‘two against three’, ‘three against four’. A few years later, Sachs (1953) and Cooper and Meyer (1960) also use the terms consonance and dissonance with respect to metre, but only in passing (see also Beauvillain, 1983, and Handel, 1984).

9 According to the results of a number of studies, the left cerebral hemisphere is involved in rhythm processing (Brust, 1980; Gordon & Bogen, 1974; Mavlov, 1980; Polk & Kertesz, 1993; Robinson & Solomon, 1974; Vuust et al., 2005). However, other studies have asserted that the processing of rhythm is not clearly lateralized (Peretz, 1990; Peretz & Morais, 1980).

10 Importantly, the perception of the pulse does not necessarily correspond to an auditory stimulus actually being presented; rather, it emerges throughout the temporal development from certain regularities that are cognitively inferred rather than instantaneously perceived. In other words, if we perceive a musical excerpt at 60 bpm, this does not imply that we perceive 60 “auditory cues” per minute.

11 Evidence concerning non-human primates (i.e., rhesus monkeys) has failed to prove the ability to detect the beat in music, thus supporting that beat induction (the cognitive mechanism that supports the perception of a regular pulse from a varying rhythm) is species-specific and absent in nonhuman primates (e.g., Honing et al., 2012).

12 In Western music, the beat-period relationship is typically expressed using integer ratios (e.g., 1 strong pulse every 2, i.e., 2:1, 1 accented pulse every 3, 3:1).

13 In the study by Occelli et al. (2009), the authors used a forced-choice task in which participants had to determine whether pairs of stimuli had the same versus different frequency. Stimuli were presented within the same sensory modality (i.e., audition or touch) or crossmodally (i.e., one in audition and the other in touch). The results showed that participants’ ability to match stimuli correctly is significantly higher in the unimodal auditory condition than unimodal tactile and crossmodal conditions.

14 Macaques are also able to produce rhythmic movements with proper tempo matching during a synchronization–continuation task (Merchant & Honing, 2014; Zarco et al., 2009; for a review, see Merchant et al., 2015). Unlike humans, however, these primates do not show an advantage for synchronizing with an acoustically rather than a visually induced beat (Zarco et al., 2009).

15 The tactile stimuli were delivered to the participant’s back, an infrequent location for stimulation in the literature on sensorimotor synchronization.

16 Intriguingly, pain is often perceived as having a pulsatile quality, described as ‘throbbing pain’ (see Mirza et al., 2012; Mo et al., 2013). However, in this review, we focus on exteroceptive signal timing, excluding interoceptive temporal perception phenomena such as throbbing pain or heartbeat awareness.

17 The perception of abstract amodal properties in sensory stimuli, namely visual and auditory, has been demonstrated in infants with respect to dimensions such as continuity/discontinuity and ascendency/descendency (Wagner et al., 1981).

18 One might hypothesize that the brain compensates for differences in skin receptor density to maintain perceptual constancy (e.g., Taylor-Clarke et al., 2004), as it appears to partially do to compensate for differences in arrival time (see Bergenheim et al., 1996).

19 While talking of ‘hearing visual rhythms’, they more cautiously note that they are really only justified in talking about temporal structure (see Sect. "Open questions and future directions").

20 Shams et al. (2000, 2002) demonstrated what the authors describe as a visual illusion induced by sound, in which the presentation of a single flash is perceived as multiple flashes when it is accompanied by multiple beeps.

21 Two recent studies by Makarov and colleagues (2024a, b) used a ‘foraging task’ in order to investigate the effect of audio-visual synchrony on a visual search paradigm. Foraging tasks involve arrays of items where multiple targets from more than a single category (e.g., red and green lines) are presented in amongst distractor items (e.g., blue and yellow lines) and observers have to select the targets (e.g., moving a mouse cursor) and avoid the distractors. In a series of three experiments, Makarov and colleagues (2024a) demonstrated that there was a large benefit of visual synchrony (i.e., the target items changing colour in synchrony) but no additional benefit for sound synchronization (i.e., a sound occurring in synchrony with colour changes). While providing evidence that visual synchronization can guide attention during multiple target foraging, the results showed no additional benefit for sound synchrony, even when the foraging task was quite difficult. In a similar task using rotating circles, Makarov et al. (2024b) found that both an auditory cue and a vibrotactile pulse used to indicate the rate of target rotation effectively reduced target detection times. These results suggest that, while crossmodal synchrony might improve participants’ performance, such an effect depends on the nature of the visual search task.

22 The results of the study by O’Leary and Rhodes (1984) demonstrated a bidirectional influence between vision and audition in a task involving perceptual organization.

23 Kubovy and Van Valkenburg (2001) doubt that such trans-modal Gestalts exist outside of the special case of audiovisual speech perception. Note here how, at the time they were writing, the only known example of a trans-modal Gestalt was from an audiovisual speech study reported by Sato et al. (2007).

24 Depending on how one defines flavour, then crossmodal perceptual grouping is widespread (see Spence, 2015, on this point). The integration of the chemical senses may be one of the only areas where crossmodal grouping is commonplace. However, note that the chemical senses have very limited temporal resolution, and hence the notion of temporal organizational structure has little relevance/meaning. That said, note that von Békésy (1964) has written on rhythmical variations accompanying gustatory stimulation observed by means of localization phenomena.

25 In this study, von Békésy aimed to demonstrate that the localization of gustatory sensations on the surface of the tongue is similar to the localization of sound sources in directional hearing or the localization of vibratory stimuli on the skin. To achieve this, he developed a method that allowed people to detect oscillations in sensation magnitude. Participants received stimulation at different locations on the tongue with a variable temporal gap. Different taste solutions (e.g., saline, sucrose, acidic and quinine sulfate) were used across several experiments, as well as water to rinse the tongue. When there was no time difference between the stimuli, the sensation was localized midway between the two stimuli. However, when there was a time difference as small as 1 ms, the locus of the sensation shifted almost entirely to the side that received the stimulus first, resulting in a sensation that periodically oscillated between the two loci. Although this phenomenon is somewhat related to rhythm, and one could probably talk about ‘rhythmic oscillations’, the task was not focused on rhythmic perception. Instead, it was a spatial task, with subjects asked to describe where they perceived the sensation, while no information about the temporal structure of the stimulation was asked. Therefore, while this study might have potential implications for studying rhythms in gustation, the results do not seem to have direct implications for understanding rhythmic perception in taste.

26 See Wilson (2023) for a philosophical discussion of temporality in olfaction.

27 Obrist and her colleagues (2014) discuss the temporal dynamics of the five basic tastes (i.e., sweet, sour, salty, bitter and umami). However, the focus in this case is on the temporal unfolding of these taste sensations rather than the emergence of a repetitive structure from the tasting experience.

28 These values might be affected by additional factors, such as the number of stimuli presented to the perceiver.

29 It takes approximately 100–150 ms for odors presented to the nasal mucosa to permeate through the mucus membrane (Hummel et al., 1996), as compared to about 40 ms for visual stimuli to be transduced at the retina (Spence & Driver, 1997; Spence & Squire, 2003). Cooke and Myin (2011) provokingly discuss the possibility of perceiving something like musical trills in olfaction, i.e., the quick alternation between two different smells, such as tobacco and rose. They ask: “Would the individual odour-samples, the 'smell sensations' that comprise the trill, be identical in quality to the temporally extended smell experiences to which we are accustomed, just briefer? Or would such extreme brevity affect their quality? When we try to imagine such an olfactory trill, is it really possible to imagine complete samples of smell-experience within it, or is there some tension here which forces us to alter the character of the experience so that it is no longer truly olfactory? Is trilled smell conceivable?” (p. 59).

30 For a philosophical reflection on the (im)possibility of rapidly presented stimuli – such as analogs of musical trills – in the sense of smell, see Cooke and Myin (2011). However, one should also note that superior temporal discrimination abilities in vision have typically been assessed at the fovea, while performance is likely to differ in the periphery (e.g., McKee & Taylor, 1984).

31 It is also worth noting that the term ‘rhythm’ is frequently used in aesthetics, visual arts and architecture (e.g., Cheyne et al., 2019; Thapa, 2017; Vara Sánchez, 2023). For instance, it may refer to how different elements structure the composition of a painting or a building. An observer might encounter a sequence of elements, such as columns. However, the experience of these artworks lacks the sequential nature and clear timing of presentation that is inherent in rhythm, as it ultimately depends on the observer’s active exploration (Gaiger, 2018).

32 In music, a trill is a rapid alternation between two adjacent notes, typically a semitone or whole tone apart. Trills are used to add expressiveness and embellishment to a piece.
